# The effects of advanced age on primary total knee arthroplasty: a meta-analysis and systematic review

**DOI:** 10.1186/s12877-016-0215-4

**Published:** 2016-02-10

**Authors:** Ethan F. Kuperman, Marin Schweizer, Parijat Joy, Xiaomei Gu, Michele M. Fang

**Affiliations:** Department of Internal Medicine, University of Iowa, Carver College of Medicine, SE 622 GH 200 Hawkins Drive, Iowa City, IA 52242 USA; Center for Comprehensive Access and Delivery Research and Evaluation, Iowa City VA Health System, Iowa City, IA USA; Department of Epidemiology, College of Public Health, University of Iowa, Iowa City, IA USA; Health Sciences Clinical Education Librarian, Hardin Library of the Health Sciences, University of Iowa, Iowa City, IA USA

**Keywords:** Knee arthroplasty, Aged, Postoperative complications, Mortality, Systematic literature review

## Abstract

**Background:**

Total knee arthroplasty is an effective treatment when nonsurgical treatments fail, but it is associated with risk of complications which may be increased in advanced age. The purpose of this study was to quantify age-related differences in perioperative morbidity and mortality after total knee arthroplasty through systematic review of existing literature.

**Methods:**

PubMed, the Cochrane database of systematic reviews, Scopus, and clinicaltrials.gov, were queried for relevant studies that compared primary total knee arthroplasty outcomes of mortality, myocardial infarction (MI), deep vein thrombosis (DVT), pulmonary embolism (PE) and functional status, of geriatric patients (>75 years old) with a younger control group (<65 years old). Pertinent journals and reference lists were hand searched. Eligibility criteria included all articles except case reports, meta-analyses, and systematic reviews. Two authors independently extracted data from each paper. Article quality was assessed using the Newcastle-Ottawa Scale.

**Results:**

Twenty-two studies were included. Geriatric patients had higher rates of mortality, MI, DVT, and length of stay in older compared to younger patients, however the absolute magnitude of these increases were small. The increase in mortality may have reflected decreased life expectancy in the geriatric populations as opposed to mortality specifically due perioperative risk. There were no differences in PE incidence and improvement in pain and functional status was equal in older and younger patients. Existing studies were limited by non-randomized patient selection, as well as variation in definitions and methodology.

**Conclusions:**

Existing data supports offering primary total knee arthroplasty to select geriatric patients, although the risk of complications may be increased. Much of the data was of poor quality. Future prospective studies are needed to better identify risks and benefits of total knee arthroplasty so that patients and surgeons can make informed decisions.

**Electronic supplementary material:**

The online version of this article (doi:10.1186/s12877-016-0215-4) contains supplementary material, which is available to authorized users.

## Background

Total knee arthroplasty (TKA) is a common and effective procedure for the treatment of end-stage osteoarthritis of the knee. Patients undergoing TKA are primarily geriatric and in the US 75 % of TKA were performed on Medicare beneficiaries [1]. Cram et al. found that annual TKA volume among Medicare enrollees increased 161 % and per capita utilization of TKA increased 99.2 % between 1991 and 2010 [2]. This increase reflects the observed improvements in pain, function, and independence in an elderly population and increasing acceptance of invasive treatment [3, 4]. Four of five patients undergoing total knee arthroplasty are satisfied with their knee replacement postoperatively [5].

However, both risks and costs may be increased in geriatric patients with comorbidities, which may lead to fewer elective orthopedic procedures. Patients aged 85 years or older are 41 % less likely to receive TKA than their younger counterparts [1]. Patients who did not undergo surgery were older and almost half of these patients were not offered TKA [6]. Prior investigations into the effects of age on various surgeries have found mixed associations [7–11], and the impact of age on candidacy for TKA is undefined. A better understanding of the risks and benefits of TKAs in this population would allow for more appropriate patient selection.

In order to clarify the effect of age on total knee arthroplasty, we systematically reviewed and evaluated studies from 1990–2015 that investigated the association of age on surgical outcomes.

## Methods

### Search strategy

This systematic literature review was conducted according to the PRISMA checklists [12]. As a review of previously published data, it was exempt from review by the local Institutional Review Board. We included all research studies that assessed clinically important outcomes of mortality, venous thromboembolism (pulmonary embolism and/or deep vein thrombosis), myocardial infarction, length of stay, and functional outcomes on geriatric patients undergoing primary total knee arthroplasty. Functional outcomes included all studies with either quantified changes in either self-reported or performance-based pain, mobility, or other symptoms.

A health sciences librarian (GX) conducted a structured literature review using the PubMed database, Scopus database, and the Cochrane database of systematic reviews for articles published from January 1, 1990 to December 16, 2015 with keywords and medical subject heading (MeSH) terms seen in Additional file 1. We reviewed the reference lists of retrieved articles to identify studies that were not obtained from the primary literature searches. Additional studies were identified through Clinicaltrials.gov.

### Inclusion and exclusion criteria

Studies were included if they met the following criteria: published in English and grouped patients by age compared to a reference younger group in patients who underwent primary total knee arthroplasty (TKA). We excluded studies if they were case reports, commentaries, guidelines, meta-analyses, systematic literature reviews, editorials, animal studies, studies that did not separate outcomes for TKA and total hip arthroplasty, pediatric studies, reviews, and those that did not contain the outcomes of interest. We excluded patients undergoing simultaneous bilateral knee replacements, as these patients are typically more carefully selected by surgical providers and there is evidence of increased complication rates [13]. Knee revision arthroplasties were also excluded as a greater fraction of these procedures are urgent and the complication rate differs significantly from primary knee procedures [2].

### Data extraction and risk of bias assessment

Two authors independently screened the titles and abstracts from 3851 citations to assess whether they met the inclusion or exclusion criteria. If the screeners did not agree whether the study was relevant, the third author reviewed the article in detail. After this initial screening, at least two of the three reviewers independently evaluated full-text articles in detail (PJ, EK, MF) and abstracted data for each article. The first author reviewed all inconsistent assessments and the reviewers resolved their disagreements by consensus.

The reviewers abstracted data from the study papers on year of publication, study design, older age group age range, younger age group age range, mortality among the older and younger group, incidence of myocardial infarction in the older and younger group, incidence of venous thromboembolism in the older and younger group, length of stay, and functional outcomes. The investigators used the Newcastle-Ottawa risk of bias tool to assess the quality of each article [14].

### Statistical analysis

Meta-analysis was performed using unadjusted data extracted from each article. We employed random-effect models to obtain pooled odds ratio estimates, using Microsoft Excel 2007 and the Cochrane Review Manager (RevMan) version 5.3 [15]. To assess heterogeneity, we used the Cochran Q statistic and the *I*^*2*^ statistic.

For length of stay and functional data qualitative terms were used to describe either a relative increase or decrease in number of days stayed in the hospital of older patients relative to younger patients and improved, worse, or no difference in functional outcomes of older patients relative to younger patients. *P* values <0.05 were determined a priori to be statistically significant. Due to the heterogeneous assumptions used in multivariate analyses, we report only univariate analyses.

## Results

**Fig. 1:**
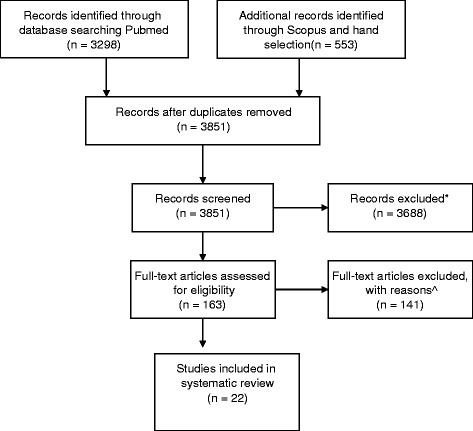
PRISMA 2009 flow diagram on selection of articles for effect of age on outcomes of TKA. *244 not in English, 3174 rejected by both initial reviewers for being irrelevant- did not address TKA as primary problem different surgical tech or med tx being compared, not knee, 274 Outcomes not addressed- eg not dvt/mortality/ssi/MI, 212 TKA bilateral or mixed with hip and knee, 1 qualitative study, 12 unavailable articles, review articles. ^49 studies did not separate hip and knee patients, 17 did not look at the outcome of interest, 4 were review articles, 60 did not study the effect of age, 8 did not have a control younger group, and 3 studies did not look at TKA

Figure 1 summarizes the search and review process. Among the 163 articles that were reviewed in detail, 22 studies on independent populations reported data that contributed to the systematic review (see Additional file 2). Some articles contained more than 1 outcome of interest. Most articles defined older patients as greater than 80 years old. The average Newcastle Ottawa score was 6, signifying moderate quality studies.

### Mortality

**Fig. 2:**
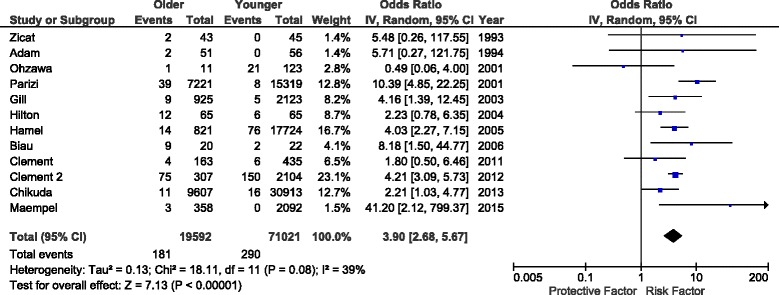
Impact of age group on mortality after TKA

Twelve studies reported deaths after elective total knee arthroplasty, with findings summarized in Fig. 2 [16–27]. There was significantly increased mortality in older patients (OR 3.90, 95 % CI 2.68–5.67), however absolute perioperative mortality was less than 1 %. There was heterogeneity in mortality definition, varying from in-hospital mortality to mortality several years post procedure. Due to this variance, we did not perform a statistical comparison of the absolute mortality rates. Although the *P* value was <0.001, the *I*^*2*^ was 39 % demonstrating moderate statistical heterogeneity. A symmetrical distribution on funnel plot suggested no publication bias. There was a statistically significant increase in mortality among the geriatric population in 7 of the 12 studies [19, 20, 22, 23, 25–27], and a non-significant trend towards increased mortality in 4 of the remaining 5 [16, 17, 21, 24]. One outlying study showed a non-significant trend towards decreased mortality in the geriatric population [18].

### Myocardial infarction

**Fig. 3:**
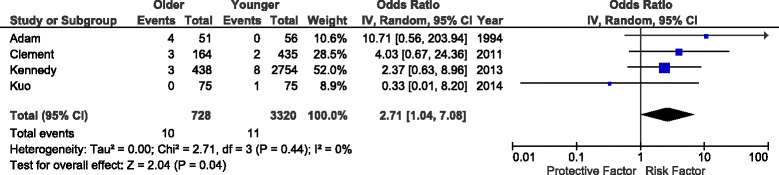
Impact of age group on perioperative MI after TKA

Five studies [16, 17, 24, 28, 29] reported MI or other cardiovascular complications after elective total knee arthroplasty in geriatric and control populations (Fig. 3). There was a significant and uniform increase in myocardial infarction in the older population (OR 2.71, *P* = 0.04, *I*^*2*^ = 0 %). Although OR was relatively consistent across studies, there was a large difference in absolute rates, varying from 0 % to nearly 8 %, suggesting significant variation in study protocol and patient population.

### Deep Vein Thrombosis

**Fig. 4:**
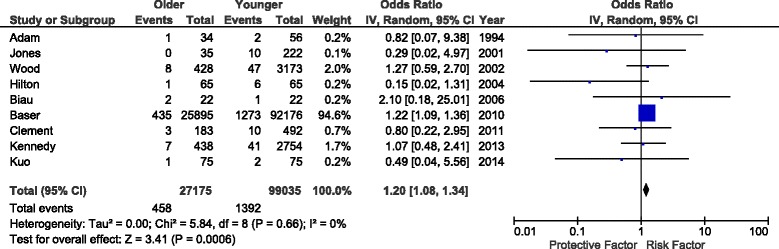
Impact of age group on perioperative DVT after TKA

Nine studies [17, 21, 23, 24, 28–32] reported deep vein thrombosis (DVT) as a complication after total knee arthroplasty (Fig. 4). The majority used ICD-9 codes or chart review and defined DVT as a complication during the hospital stay [17, 21, 24, 28, 29, 32]. The remaining studies evaluated symptomatic DVT between 3 and 12 months postoperatively. Although meta-analysis identified a small increase in DVT rate (OR 1.20, *P* 0.0006, *I*^*2*^ = 0 %), 94.6 % of the weight was given to a single study [32], so results should be interpreted cautiously.

### Pulmonary embolism

**Fig. 5:**
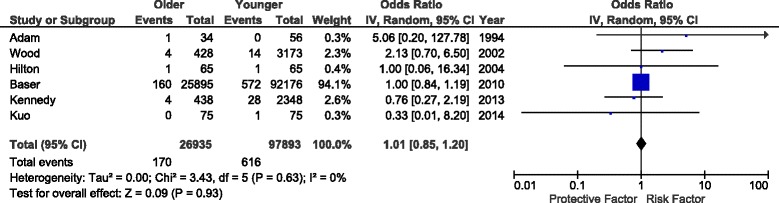
Impact of age group on perioperative PE after TKA

Six studies [17, 21, 28, 29, 31, 32] reported pulmonary embolism (PE) as a complication after total knee arthroplasty (Fig. 5). The rate of PE was identical in older and younger populations (OR 1.01, *P* = 0.93, *I*^*2*^ = 0 %). Once again, a single study was responsible for >90 % of the weight of the analysis, so results should be interpreted cautiously [32].

### Length of stay

Six studies [17, 21, 24, 29, 33, 34] provided sufficient data to calculate lengths of stay for older and younger populations (Table 1). There was uniformly increased length of stay among older patients across studies. There was also decreasing length of stay for all patients (both older and younger) in more contemporary studies, consistent with a secular trend. Due to this trend, meta-analysis was not performed.

**Table 1:** Effect of age on length of stay

Reference number	First author	Year of publication	Number of patients	LOS geriatric (95 % CI)	LOS control (95 % CI)	*P* value	Age groups
17	Adam	1994	107	22^a^	16^a^	Not reported	>75, < 75
21	Hilton	2004	130	13 (5–29)	10 (8–22)	<0.001	80–90, 60–70
24	Clement	2011	598	8.3^a^	6.2^a^	<0.001	>80, 65–74
29	Kuo	2014	150	6.1^a^	5.7^a^	0.061	>80, 65–74
30	Jones	2001	257	7 (5–9)	6 (4–8)	0.04	>80, < 80
33	Vincent	2006	424	10.6^a^	8.7^a^	0.004	>70, < 60
34	Smith	2008	2096	11.8^a^	8.8^a^	<0.001	>80, 60–69

^a^Confidence interval not reported

### Functional data

Post-operative functional outcomes were compared in eleven studies [16, 17, 21, 23, 24, 27–29, 35–37] and is summarized in Table 2. There was variation in follow-up period and the methodology used to assess improvement. These included subjective, self-reported scales such as the Oxford Knee Score [24, 28, 37], and EQ-5D [38] as well as instruments containing objectively measured data such as the Knee Score [17, 23, 35]. Due to the variation in the measurement scales it was not feasible to statistically combine these results. When comparing the geriatric to younger patients undergoing TKA, six studies reported no statistically significant differences in post-operative functional status [16, 17, 21, 23, 24, 27, 29, 36]. Three studies [28, 35, 37] reported statistically worse scores in older patients. Some studies reported more dissatisfaction than the geriatric among the younger patients with regards to functional improvement scores [21] and pain perception [16, 21] In one study patients aged < 55 years exhibited a lower mean level of satisfaction compared with all other age groups (*p* < 0.031) [37]. Younger patients in one study complained more about their prostheses [21].

**Table 2:** Effect of age on functional scores postoperatively

Reference Number	First Author	Year of Publication	Number of participants	Functional score comparison	Age groups	Outcome
16	Zicat	1993	90	No difference	≥80, 65–69	Hospital for Special Surgery Score
17	Adam	1994	107	No difference	≥75, <75	Knee Score
21	Hilton	2004	130	No difference	80–90, 60–70	Knee Society Score
23	Biau	2006	42	No difference	≥85, 65–75	Knee Score
24	Clement	2011	598	No difference	80–92, 65–75	Oxford Knee Score
27	Maempel	2015	2050	No difference	≥80, < 75	Knee Society Score
28	Kennedy	2013	3192	Geriatric worse	≥80, <79	Oxford Knee Score
29	Kuo	2015	150	No Difference	≥80, 65–74	Knee Society Score
35	Laskin	1999	1634	Geriatric worse	≥85, 62–85	Knee society score
36	Hernandez	2006	218	No difference	75–98, <75	Hospital for Special Surgery Score
37	Williams	2013	238	No difference	≥85, 55–84	Oxford knee score

## Discussion

The currently available literature provides limited information on the potential safety and efficacy of knee replacement in the geriatric population. This information is limited by the ability to uniformly define either our population of interest or the optimal endpoints to measure. Although morbidity may be increased in this population, the absolute incidence of each endpoint is small while benefits appear to be uniform across the age groups. Thus, elective knee arthroplasty could be beneficial in select geriatric individuals.

There was variation in methodology, including the definition of age groups and the duration of follow-up, however the weight of available evidence suggests increased mortality for geriatric patients in both the immediate post-operative period as well as several years following TKA. Although mortality is a principle indicator of patient safety, it has limited application as a marker for total knee arthroplasty. Short-term mortality after TKA is less than 0.5 % [19, 38]. Mahomed, et al. in a study of Medicare recipients in 2000 found that there was a 90-day mortality rate of 0.6; (95 % confidence interval, 0.6–0.7) which was fewer deaths than would be expected in the unselected Medicare population who did not undergo total joint replacement [39]. Geriatric patients were enrolled for elective surgery in all identified studies, which introduces the potential for substantial selection bias in sampling. For knee replacements in the general population, mortality returns to baseline levels approximately one month after surgery [40]. It is not clear that this trend would apply to the geriatric population, but over several years it seems likely that baseline mortality trends would dominate. The life expectancy for an 85-year-old patient in the US is approximately 6 years [41]. Examining mortality rates 10 years postoperatively in this population does not make clinical sense.

Second, there is limited evidence of an increased complication rate for TKA performed in the geriatric population. In addition to the meta-analysis above, two large studies that did not meet inclusion criteria with over 200,000 patients reported statistically significant increased rates of acute MI in short term follow-up periods of six weeks (≥80 years [HR 8.20 (2.38–28.22)], 60–79years [HR 2.55 (0.77–8.42)] [42] and ninety days [RR 5.0 (2.8–8.6) >90 years relative to 65–69 years [39]. In contrast, there was no significant increase in DVT or PE in older patients despite increased incidence of common risk factors such as cancer, congestive heart failure, and impaired mobility [43]. This may have been due to the low incidence of postoperative DVT, as low as 0.4 % in Medicare patients receiving TKA using prophylactic strategies [2]. Other potential explanations include more aggressive perioperative DVT prophylaxis in geriatric patients with comorbidities, as well as selection bias towards patients with minimal comorbidities in elective TKA. DVT prophylaxis techniques were not mentioned in the majority of studies. Hilton [21] stated that all patients used antithrombotic stockings and 43 % used heparin subcutaneously twice daily. Biau [23] stated that “prophylaxis was given to everyone” and Clement [24] stated that DVT prophylaxis was given based according to the Scottish Intercollegiate Guidelines. Kennedy mentioned that the elderly patients may have had more thorough venous thromboembolism prophylaxis rates [28].

Both geriatric and control patients had similarly high satisfaction with their joints ranking similar scores for overall satisfaction and improvement in functional status. Compared to the population norm for a particular age group, patients aged 75 years and over improved significantly, becoming similar to population norms for this age [44]. Studies have shown that geriatric have similar to worse functional scores at baseline compared to younger patients [2] implying that geriatric patients may delay surgery longer or they are being denied surgery that younger patients would have received. Despite this, patients who underwent total joint arthroplasty had significantly improved WOMAC scores compared with those who did not undergo surgery, however, 45 % of those who did not have surgery were not offered a surgical option [6]. The other 55 % felt that pain and complications of surgery and needing someone to care for them after surgery were hurdles to surgery [6]. Although the direct cost of the operation may be increased due to the greater LOS, this cost may need to be weighed not against younger controls but against the increased caregiver needs and loss of independence in those patients treated without invasive therapy. Further studies may be necessary to better define the cost-effectiveness of the procedure in the geriatric population.

### Limitations

This review was limited due to the nature of the extant available literature. Definitions varied, not only the definition of “geriatric” but also of mortality, myocardial infarction, DVT, functional recovery, which complicate efforts to synthesize the data into a single answer. The vast majority of studies reviewed for this article compared a selected geriatric population to matched, younger controls. There is limited data describing how these patients were selected, and the results may not be applicable to unselected geriatric patients presenting for initial evaluation. Many of the studies were done in the 1990’s making it difficult to compare to contemporary surgeries, as there have been significant changes in intraoperative and perioperative management since that time.

## Conclusions

In conclusion, the available data suggests that selected geriatric patients face similar risk to younger patients undergoing elective TKA, with comparable improvements in functional status. In patients with good functional status and minimal medical comorbidities, age alone should not contraindicate knee arthroplasty. Future, dedicated investigations are need improve selection criteria.

## Additional files

Additional file 1:
**Pubmed search strategy.** (DOCX 13 kb) Additional file 2:
**List of all articles meeting inclusion criteria.** (DOC 21 kb)

## Abbreviations

DVT: deep vein thrombosis
LOS: length of stay
MI: myocardial infarction
PE: pulmonary embolism
TKA: total knee arthroplasty
