# Supplementary material for: The effects of advanced age on primary total knee arthroplasty: a meta-analysis and systematic review
Source: BMC Geriatr. 2016 Feb 10;16:41. doi: 10.1186/s12877-016-0215-4 (PMC4750247; doi:10.1186/s12877-016-0215-4)
Supplement: Additional file 1: — Pubmed search strategy. (DOCX 13 kb) [file 12877_2016_215_MOESM1_ESM.docx]

Additional file 1

Pubmed search strategy

| 1. "Arthroplasty, Replacement, Knee"[Mesh] OR knee arthroplast*[text word] OR knee replace*[text word] |
| --- |
| 1. Prognosis[Mesh] OR “Risk assessment”[Mesh] OR "Incidence"[Mesh] OR "Mortality"[Mesh] OR “mortality" [Subheading] OR "Risk Factors"[Mesh] OR "Treatment Outcome"[Mesh] OR "Survival Rate"[Mesh] OR "Follow-Up Studies"[Mesh] OR "adverse effects" [Subheading] OR "Death"[Mesh] "Cohort Studies"[Mesh] OR "Forecasting"[Mesh] OR Prognos*[text word] OR Risk assessment[text word] OR Incidence[text word] OR Mortality[text word] OR Risk Factor*[text word] OR Outcome*[text word] OR Surviv*[text word] OR Follow-Up [text word] OR Follow up[text word] OR adverse effect*[text word] OR Death[text word] OR Cohort*[text word] OR Forecast*[text word] OR "Survival Analysis"[Mesh] OR Survival Analys*[text word] OR rejection*[text word] OR “contraindications”[subheading] OR contraindicate*[text word] OR "Length of Stay"[Mesh] OR length of stay[text word] OR readmission*[text word] OR "Patient Readmission"[Mesh] OR "Activities of Daily Living"[Mesh] |
| 1. "Age Factors"[Mesh] OR Age[text word] OR aged[text word] OR ages[text word] OR elderly[text word] OR senior*[text word] OR medicare[text word] |
| 1. "Retrospective Studies"[Mesh] OR Cohort Stud*[text word] OR Longitudinal Stud*[text word] OR Follow-Up Stud*[text word] OR followup stud*[text word] OR follow up[text word] OR Prospective Study[text word] OR prospective studies[text word] OR Retrospective[text word] OR incidence stud*[text word] |
| 1. #1 AND #2 AND #3 AND #4 |

Scopus search strategy

**Use AND to connect the 4 searches below**

#1

“knee arthroplast*” OR “knee replace*” OR “Knee Prosthesis” OR “Knee Prostheses” OR “knee implant*” OR “Knee Endoprosthesis” OR “knee Endoprostheses”

#2

“Age factor” OR age OR elderly OR medicare OR senior OR gerontology OR geriatrics OR “age related” OR “older adult” OR “older patient” OR “aged”

#3

“Retrospective Study” OR “Cohort Study” OR “Longitudinal Study” OR “Follow-Up Study” OR “followup study” OR “prospective study” OR “incidence study”

#4

“Deep vein thrombosis” OR “Venous thromboembolism” OR “pulmonary embolism” OR “Pulmonary Thromboembolism” OR “myocardial infarct” OR “heart attack” OR “Surgical Wound Infection” OR “Postoperative Wound Infection” OR Sepsis OR Septicemia OR Pyaemia OR Pyemia OR Hemorrhage OR haemorrhage OR bleeding OR Mortality OR “Death Rate” OR “Case Fatality Rate” OR “survival rate” OR Survivorship OR “Mean Survival Time” OR “survival analysis” OR “recovery of function” OR “Function Recovery” OR “Functional improvement” OR “quality of life” OR “life qualities” OR SF-36 OR “summary score” OR “functional competence” OR “Health Status Indicator” OR “health status index” OR “health risk appraisal” OR “Patient Readmission” OR “Length of stay” OR “stay length” OR “hospital stay” OR “discharge disposition” OR Rehabilitation OR Habilitation OR “Activities of Daily Living” OR “Activity of Daily Living” OR “chronic limitation of activity” OR “Postacute care” OR “post-acute care” OR “extended care facility” OR “Skilled Nursing Facility”
