# Supplementary material for: The effects of advanced age on primary total knee arthroplasty: a meta-analysis and systematic review
Source: BMC Geriatr. 2016 Feb 10;16:41. doi: 10.1186/s12877-016-0215-4 (PMC4750247; doi:10.1186/s12877-016-0215-4)
Supplement: Additional file 2: — List of all articles meeting inclusion criteria. (DOC 21 kb) [file 12877_2016_215_MOESM2_ESM.doc]

Additional file 2: List of all articles meeting inclusion criteria.

| Reference number | First Author | Year of publication | Study design | Number of patients | Definition of Elderly | Definition of control | Newcastle-Ottawa scale | Endpoints reviewed |
| --- | --- | --- | --- | --- | --- | --- | --- | --- |
| 16 | Zicat | 1993 | Matched case control | 90 | >80 | 65-69 | 9 | Mortality, MI, functional data |
| 17 | Adam | 1994 | Unmatched case control | 125 | 75-84 | 47-74 | 5 | Mortality, MI, DVT, PE, LOS, functional data |
| 18 | Ohzawa | 2001 | Cohort | 134 | >75 | <74 | 7 | Mortality |
| 19 | Parvizi | 2001 | Cohort | 22540 | >70 | <70 | 7 | Mortality |
| 20 | Gill | 2003 | Cohort | 3048 | 75-94 | <74 | 7 | Mortality |
| 21 | Hilton | 2004 | Unmatched case control | 130 | 80-90 | 60-70 | 5 | Mortality, DVT, PE, LOS, functional data |
| 22 | Hamel | 2005 | Cohort | 18545 | >80 | <79 | 6 | Mortality |
| 23 | Biau | 2006 | Matched case control | 42 | >85 | 65-75 | 8 | Mortality, DVT, functional data |
| 24 | Clement | 2011 | Unmatched case control | 677 | 80-92 | 65-75 | 7 | Mortality, MI, DVT, LOS, functional data |
| 25 | Clement | 2012 | Cohort | 2409 | >80 | <79 | 6 | Mortality, LOS |
| 26 | Chikuda | 2013 | Cohort | 37001 | >80 | 65-79 | 7 | Mortality |
| 27 | Maempel | 2015 | Cohort | 2450 | >80 | <75 | 8 | Mortality, functional data |
| 28 | Kennedy | 2013 | Unmatched case control | 3192 | >80 | <79 | 7 | MI, DVT, PE, functional data |
| 29 | Kuo | 2014 | Matched case control | 150 | >80 | 65-74 | 6 | MI, DVT, PE, LOS, functional data |
| 30 | Jones | 2001 | Cohort | 257 | >80 | <80 | 7 | DVT, LOS, functional data |
| 31 | Wood | 2002 | Cohort | 3601 | >70 | <69 | 7 | DVT, PE |
| 32 | Baser | 2010 | Cohort | 117354 | >80 | 65-79 | 7 | DVT, PE |
| 33 | Vincent | 2006 | Cohort | 424 | >70 | <60 | 7 | LOS |
| 34 | Smith | 2008 | Cohort | 2096 | >80 | 60-69 | 7 | LOS |
| 35 | Laskin | 1999 | Cohort | 1634 | >85 | 62-85 | 7 | functional data |
| 36 | Hernandez | 2006 | Cohort | 218 | 75-98 | <75 | 7 | functional data |
| 37 | Williams | 2013 | Cohort | 238 | >85 | 55-84 | 6 | functional data |
